# *Zymomonas mobilis*: a novel platform for future biorefineries

**DOI:** 10.1186/1754-6834-7-101

**Published:** 2014-07-02

**Authors:** Ming Xiong He, Bo Wu, Han Qin, Zhi Yong Ruan, Fu Rong Tan, Jing Li Wang, Zong Xia Shui, Li Chun Dai, Qi Li Zhu, Ke Pan, Xiao Yu Tang, Wen Guo Wang, Qi Chun Hu

**Affiliations:** 1 Biogas Institute of Ministry of Agriculture, Biomass Energy Technology Research Centre, Section 4-13, Renming Nanlu, Chengdu 610041, P. R. China; 2 Key Laboratory of Development and Application of Rural Renewable Energy, Ministry of Agriculture, Chengdu 610041, P. R. China; 3 Institute of Agricultural Resources and Regional Planning, Chinese Academy of Agricultural Sciences, Beijing 100081, P. R. China

**Keywords:** *Zymomonas mobilis*, platform, biorefinery, biofuel, building block chemical

## Abstract

Biosynthesis of liquid fuels and biomass-based building block chemicals from microorganisms have been regarded as a competitive alternative route to traditional. *Zymomonas mobilis* possesses a number of desirable characteristics for its special Entner-Doudoroff pathway, which makes it an ideal platform for both metabolic engineering and commercial-scale production of desirable bio-products as the same as *Escherichia coli* and *Saccharomyces cerevisiae* based on consideration of future biomass biorefinery. *Z. mobilis* has been studied extensively on both fundamental and applied level, which will provide a basis for industrial biotechnology in the future. Furthermore, metabolic engineering of *Z. mobilis* for enhancing bio-ethanol production from biomass resources has been significantly promoted by different methods (i.e. mutagenesis, adaptive laboratory evolution, specific gene knock-out, and metabolic engineering). In addition, the feasibility of representative metabolites, i.e. sorbitol, bionic acid, levan, succinic acid, isobutanol, and isobutanol produced by *Z. mobilis* and the strategies for strain improvements are also discussed or highlighted in this paper. Moreover, this review will present some guidelines for future developments in the bio-based chemical production using *Z. mobilis* as a novel industrial platform for future biofineries.

## Introduction

There have been growing concerns about biosynthesis of fuels, desired chemicals and materials from renewable biomass resources for limited fossil resources and associated environmental issues in the past few decades [1,2]. As model industrial or laboratory organisms, *Escherichia coli* and *Saccharomyces cerevisiae* were selected as important platforms for the purpose of desired biofuels and chemicals production via metabolic engineering [3-5]. Currently, strain optimization to utilize various feedstocks (for example, starch, sugarcane, agricultural residues, industrial waste, forest residues, energy crops, et cetera) [6,7], desired products spectrum (for example, biofuels and building block chemicals), and higher yields, which have made great progress in the past decades and provided a basis for industrial applications [1-5].

**Figure 1:**
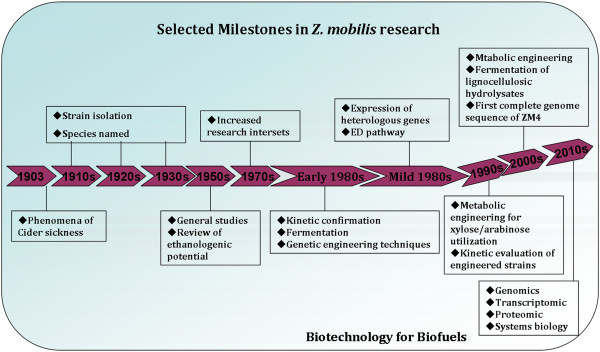
**Selected milestones in *Z. mobilis *** **research.**

As a candidate bio-ethanol producer, *Zymomonas mobilis* showed some advantages, for example, higher specific rate of sugar uptake, high ethanol yield, lower biomass production, non-requirement of controlled addition of oxygen during fermentation, et cetera [8-13]. Extensive fundamental studies on *Z. mobilis* over the last 30 years have also made this strain a promising ethanologenic organism for large-scale bio-ethanol production. On the other hand, extensive studies on different genetic techniques (including plasmid vector, expression system, transposon system, gene knockout, gene transformation, and gene function, et cetera) will help *Z. mobilis* are amenability to genetic improvement for industrial biotechnology [13]. Furthermore, strategies of strain improvement (such as conventional mutagenesis, transposon mutagenesis, adaptive laboratory evolution, and metabolic pathway engineering, et cetera), and different value-added bio-products have also been paid more and more attention in the past 20 years. Importantly, genomics and transcriptomic of *Z. mobilis* have also been developed since 2005, which will aid future metabolic engineering and synthetic biology in strain improvement for industrial applications [14]. Selected milestones in *Z. mobilis* research are summarized in Figure 1.

Currently, three subspecies (subsp.) of *Z. mobilis* have been found, including *Z. mobilis* subsp. *mobilis*, *Z. mobilis* subsp. *pomaceae* and *Z. mobilis* subsp. *Francensis*[15-19]. All strains have also been summarized in the Ph D thesis of So Lok-yan (University of Hong Kong) and other review articles [19]. Among these strains, ATCC 31821 (ZM4), ATCC 10988 (ZM1), ATCC29191 (ZM6), CP4, and NCIMB 11163 from *Z. mobilis* subsp. *mobilis*, ATCC 29192 from *Z. mobilis* subsp. *pomaceae*, which were well-charcterized by previous studies on the level of physiology, biochemical, fermentation, genetics, metabolism, and *omics*. These strains are regarded as a model organism in *Z. mobilis* research or industrial applications.

In general, *Z. mobilis* may play a critical role as a novel platform in industrial biotechnology for the development of a green replacement for petrochemical products. In this paper, we review some critical research progress on *Z. mobilis* for its use as a platform for the production of ethanol and other buck chemicals from biomass.

## Review

### Genetic background of *Z. mobilis*

Currently, general genetic tools have been developed in *Z. mobilis* since the 1980s, including native plasmids, broad host-range vectors or shuttle vectors, expression system, gene transfer, promoter, and reporter gene, as reviewed in other articles [8,11,13]. Specific gene knockout*,* genomics, and transcriptomics will be emphasised as below.

#### *Specific gene knockout*

The development of gene deletion approaches have been performed for gene function and there has also been greatly improved metabolic engineering of *Z. mobilis*. Currently, different methods, including insertional mutant, suicide plasmid-based mutant construction, site-specific FLP recombinase, fusion-PCR-based construction technique, and transposon mutagenesis, have been employed for inactivating specific genes of *Z. mobilis*. Up to date, many genes, such as pyruvate decarboxylase (*pdc*, ZMO1360), alcohol dehydrogenase (*adhB*, ZMO1596), lactate dehydrogenase (*ldhA*, ZMO1237), NADH dehydrogenase (*ndh*, ZMO1113), RNA-binding protein *Hfq* (*hfq*, ZMO0347), hydroxylamine reductase (*nha*A, ZMO0117), glucose-fructose oxidoreductase (*gfo*, ZMO0689), aldo/keto reductase (*him*A, ZMO0976), restriction-modification (R-M) systems-related gene (ZMO0028, ZMO1933, ZMO1934, ZMO1934, ZMO0575), cytochrome-related gene (*cyt*C, *cyt*B, *ctb*D, ZMO0957, ZMO1572) et cetera, which were selected as targets for improvement of some specific phenotype (summarized in Table 1).

**Table 1:** **Summary of specific gene knockout in *Z. mobilis***

**Gene inactive**	**Method**	**Description**	**References**
Extracellular sucrase gene (*sacC,* ZMO0375)	Insertional mutant	Improves levan production	[20]
Restriction-Modification	Insertional mutant or Homologous recombination	Increased transformation efficiency	[21,22]
(R-M) systems related gene (ZMO0028, ZMO1932, ZMO1933, ZMO1934, ZMO1935)			
*pdc* (ZMO1360)	Homologous recombination	Lower ethanol and lactate yield, and higher succinate concentration from glucose	[23]
*adhB* (ZMO1596)			
*ldhA*(ZMO1237)			
*him*A (ZMO0976)	Transposon mutagenesis	Reduced himA activity and increased ethanol production compared to parental strains when cultured in a mixed-sugar medium containing xylose, especially in the presence of acetate	[24,25]
*ndh* (ZMO1113)	Insertional mutant	Low respiration rate, higher cell growth and ethanol yield under aerobic conditions	[26,27]
*hfq* (ZMO0347)	pKNOCK suicide plasmid-based mutant construction	More sensitive to multiple lignocellulosic pretreatment inhibitors and hasan increased lag phase duration and/or slower growth depending upon the conditions; and verified that *hfq*playsa role in tolerance to multiple biomass pretreatment inhibitors, including acetate, vanillin, furfural, and HMF	[28]
*nha*A (ZMO0117)	Insertional mutant	Cell growth decreased under sodium acetate condition	[29]
Xylose reductase (XR, ZMO0976)	Homologous recombination	Improvement of xylose utilization	[30]
*gfo* (ZMO0689)	Site-specific FLP recombinase	Improves growth and ethanol production without formation of sorbitol as a by-product in sucrose medium, but yields opposite effects in high glucose	[31]
*gfo* (ZMO0689)	Homologous recombination (fusion-PCR-based construction technique)	Reduction of cell growth and ethanol production under osmotic, heat and ethanol stresses	[32]
*cytC*	Insertional mutant	Exhibsfilamentous shapes and reduction in growth under a shaking condition at a high temperature	[33]
*cytB* (ZMO0957), *ctdB* (ZMO1572)	Insertional mutant	Low respiration capacity when cultivated anaerobically	[34]
*psp* operon (ZMO1061-ZMO1065)	Homologous recombination (fusion-PCR-based construction technique)	Mutiple phenotypes	Our laboratory, unpublished data
Mutant library	Transposon mutagenesis	Mutiple phenotypes	Our laboratory, unpublished data

#### *Sequenced genome of different Z. mobilis strains*

Genome sequencing technology provides opportunities for fundamental insights and facilitates strain development [35]. Seo *et al*. reported the first genome sequence of *Z. mobilis* ZM4 in 2005. The complete genome of *Z. mobilis* ZM4 contains a 2,056,416-bp circular chromosome and five circular plasmids [9]. The complete genome sequence of other *Z. mobilis* strains have also been reported since 2005 [36-41]. All strains contain a circular chromosome and types of plasimd. However, genome sizes are various among these strains, ranging from 2.01 to 2.22, with two to six plasimds existing (Table 2). Although the genome of seven strains has been sequenced by different organizations, the comparative genome analysis has not been reported in public.

**Table 2:** **Genomics, transcriptome or gene expression in different *Z. mobilis *** **strains**

**Sequenced genome**^**a**^					
***Z. mobilis*** **strain**	**Accsession number**	**Description**			**References**
		**Size (Mb)**	**Plasmids**	**Protein**	
ZM4 (ATCC31821)	NC_006526.2	2.06	5	1,738	[9]
NCIMB11163	NC_013355.1	2.22	3	1,884	[36]
ATCC 29191	NC_018145.1	2.01	3	1,709	[37]
ATCC 29192	NC_015709.1	2.06	2	1,748	[38]
ATCC 10988	NC_017262.1	2.14	6	1,803	[39]
ZM401 (ATCC 31822)	Draft genome sequence	2.04	Not found	1,910	[40]
CP4 (NRRL B-14023)	NC_022900.1	2.16	5	1,840	[41]
	(CP006818.1)				
**Transcriptome or gene expression**					
ZM4 (ATCC31821)	GSE10302	Transcriptomic profiling of ZM4 during aerobic and anaerobic fermentations			[42]
	GSE37848	Expression profiling of ZM4 in response to furfural stress			[43]
	GSE39558	Transcriptomic profiling of ZM4 in response to ethanol stress			[44]
	GSE21165	Systems biology analysis of ZM4 ethanol stress responses			[45]
	GSE39466	Comparison of gene expression and mutant fitness in ZM4			Lawrence Berkeley Laboratory, unpublished data
	GSE51870	Expression data for ZM4 growing in rich and minimal media, heat-shocked, or at high ethanol			Lawrence Berkeley Laboratory, unpublished data
ZM4 (AcR)	GSE18106	Genome changes associated with *Z. mobilis* sodium acetate-tolerant mutant (AcR)			[29]
RDM-4 strain of *Z. mobilis*	GSE22355	Expression analysis of a respiration-deficient mutant of *Z. mobilis* ZM6			Faculty of Food and Nutrition, Beppu university
ZM401	Not deposited	Genome-wide transcriptomic analysis of a flocculent strain of *Z. mobilis* ZM401			[46]
ZM4 (ATCC31821)	GSE49620	Transcriptional responses of *Z. mobilis* to osmotic shock of high glucose concentration			Unpublished data, performed by Sichuan University and Biogas Institute of Ministry of Agriculture

^a^Detailed information on genome projects of *Z. mobilis*canbe accessed at the NCBI Microbial Genomes Resources database: http://www.ncbi.nlm.nih.gov/genome/?term=zymomonas+mobilisor the Genomes OnLine Database at: http://www.genomesonline.org/.

#### *Transcriptome or gene expression of Z. mobilis*

With different genome projects of *Z. mobilis* performed, further comparative genomics or global expression analysis could provide some guidelines for strain improvement in the future. Currently, many researchers are also focusing on transcriptomic profiling of *Z. mobilis* to better understand the network of gene or metabolic regulation. Especially, DNA microarray techniques or DNA sequencing have been used to identify differential gene expression under nutrition limitation, environmental stress (that is, heat stress, ethanol, furfural, et cetera). To date, there are ten datasets (including some unpublished data) from Gene Expression Omnibus (GEO) database (Table 2). For example, transcriptomic profiling of ZM4 during aerobic and anaerobic fermentations have been investigated for the first time [42]. Transcriptomic profiling of ZM4 in response to ethanol and furfural stress were also performed by our laboratory [43,44]. Integrated “omics” approach (that is transcriptomic, proteomic and metabolic) was also used for studing the molecular mechanisms of ethanol stress response in ZM4 for the first time [45]. Expression data for ZM4 growing in rich and minimal media, heat-shocked, or at high ethanol were also performed by Lawrence Berkeley Laboratory (unpublished data). Genome changes associated with *Z. mobilis* sodium acetate-tolerant mutant (AcR) was aslo reported by Yang *et al*. In this study, next-generation sequencing (NGS), comparative genomics, transcriptomics, and genetics were used to elucidate the molecular mechanism of AcR sodium acetate tolerance. Especially, a key gene, *nha*A (ZMO0119), which conferred sodium acetate (NaAc) tolerance in *Z. mobilis*[29]. ZM401 (a flocculent mutant strain of *Z. mobilis*) was also studied by using genome-wide transcriptomic technology, which provided a deep understanding for evidence related to phenotypic changes associated with its cell-cell attachment behavior. These expression data indicate that cellulose and synthesis flagella-related proteins synthesis play an important role in its special flocculent behavior in ZM401 [46]. These studies will provide insights into molecular response to environmental stress in *Z. mobilis* or help to construct more resistant strains for ethanol or other chemical production in the future. In conclusion, those transcriptomic profiling generated in these studies will likely serve as useful reference data for industrial strain development at the level of systems biology in the future.

### Strain improvement for *Z. mobilis*

#### *Strain improvement by conventional mutagenesis*

Traditionally, strain improvement was achieved mainly by mutagenesis and selection, which are still very useful in *Z. mobilis*. Currently, different mutagenesis agents, including UV light, 1-methyl-3-nitro-1-nitrosoguanidine (NTG), caffeine, ethyl methane sulfonate (EMS), et cetera, were used for *Z. mobilis* phenotype improvement. Many mutants were obtained by these mutageneses, that is, auxotrophic, ethanol and salt-tolerant, acetaldehyde-tolerant, osmotolerant, thermotolerant, sucrose-hypertolerant, acid-tolerant, fructose-negative, glucose-negative, mannitol-utilizing, levan-producing, and antibiotic-sensitive strains, et cetera (as reviewed by other authors) [8]. Among these mutants, environmental stress-tolerant mutant, and antibiotic-sensitive strains have showed some potential in industrial applications. For example, the acetate-tolerant *Z. mobilis* mutant (AcR) was generated by chemical mutagenesis and selection in the presence of acetate [47], and used as a host for constructing of engineered tolerant *Z. mobilis* strain for bio-ethanol production, that is ZM4/Ac^R^ (pZB5) [48-50].

#### *Strain improvement by transposon mutagenesis*

Transposon mutagenesis has also provided an alternate mutational approach in *Z. mobilis*. Although different transposons, including Tn*5* and Tn*10*[51], Tn*951*[52] and Tn*1725*[53], which are carried by broad host-range plasmids, have been successfully transferred into *Z. mobilis*, no transposition event have been found. Morever, Carey *et al*. first found that plasmid pGC91.14 (RP1::Tn951) was stable in *Z. mobilis* at 30°C, and the *lac* operon encoded by Tn 951 was expressed sucessfully in *Z. mobilis*[52]. Pappas *et al*. also compared of the stability of different transposable elements Tn*5*, Tn*501* or mini Mu in *Z. mobilis*, and the plasmid pULB113 (RP4::mini Mu) exhibited higher stability than others. With the help of mini Mu transposon, a large number of independent and stable auxotrophic mutants with polyauxotrophs, cysteine, methionine and isoleucine requiring-isolates were obtained [54]. The study proved that transposon mutagenesis is an extremely powerful tool for mutant construction in *Z. mobilis*[54,55]. For example, Tn5 transposon was also used for construction of recombinant strain for ethanol production [56]. Actually, there are some transposon elements in *Z. mobilis* strains*.* For example, IS5-like insertion sequence, designated IS*Zm1068*, was firstly isolated from *Z. mobilis* CP4, which was kept active in *E. coli* and led to plasmid replicon fusions [57].

#### *Strain improvement by adaptive laboratory evolution (ALE)*

Adaptive laboratory evolution (ALE) has emerged as a valuable method in metabolic engineering for strain development and optimization [58-62], and has been used successfully in model organisms such as *E. coli*[63,64] and *S. cerevisiae*[65-68]. Previous studies demonstrated that adaptation and metabolic engineering can be used synergistically for strain improvement. Recently, ALE strategy was also employed for *Z. mobilis* strain improvement. For example, an adaptive mutation procedure was developed for screening of acetic acid-tolerant *Z. mobilis*, and many adapted mutants obtained for further use in bio-ethanol production [69]. Agrawal *et al.* also used this method to select a highly efficient xylose-fermenting *Z. mobilis* strain A3 [70]. These two studies demonstrated that the ALE method might be used as a powerful metabolic engineering strategy for improving certain features of *Z. mobilis* in the future, for example, inhibitor tolerance or substrate utilization.

#### *Increase in the substrate utilization range of Z. mobilis*

**Figure 2:**
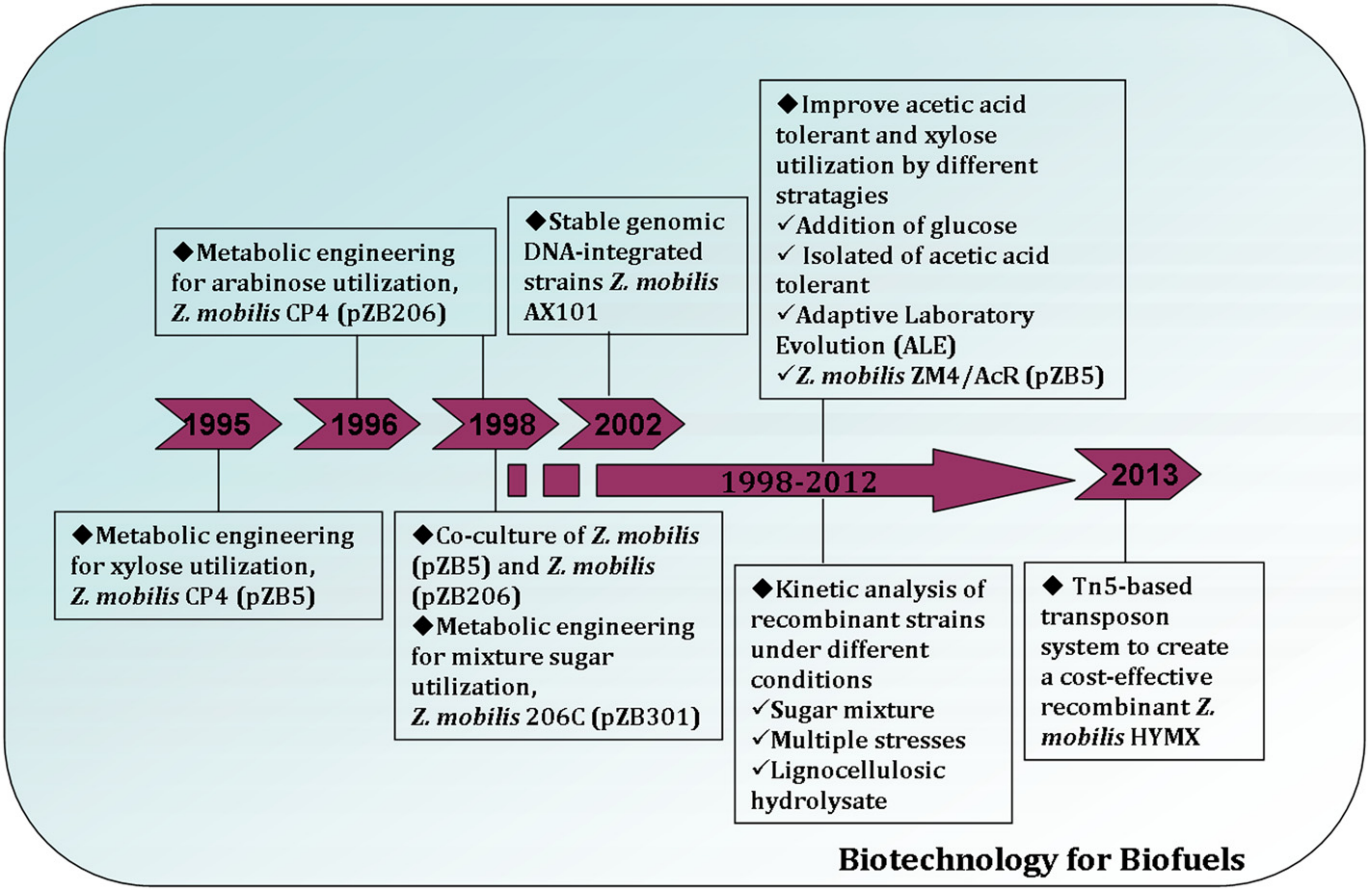
**Research history of recombinant *Z. mobilis *** **for ethanol production.**

Extensive studies or reviews on ethanol production from sugarcane, molasses, starch, and glucose by *Z. mobilis* have been performed by many authors [8,10-13,19,71,72]. Based on the consideration of some debates about food security [73], environmental degradation [74] and other issues, developing lignocellulosic feedstocks to substitute corn or sugarcane for bioenergy production will be an inevitable trend in the future [75]. Currently, recombinant *Z. mobilis* capable of simultaneous fermentation of pentose and hexose sugars from lignocellulosic hydrolysates to ethanol have been achieved since 1995. The brief research history is shown in Figure 2.

In 1995, Zhang *et al.* from the National Renewable Energy Laboratory (NREL) constructed a recombinant *Z. mobilis* CP4 (pZB5) strain by introducing two operons encoding xylose assimilation and pentose phosphate pathway enzymes from *E. coli* into *Z. mobilis* for the first time, which could ferment pentose sugar and allowing for growth on xylose with 86% ethanol yield [76]. Based on Zhang’s research, another arabinose-fermenting recombinant *Z. mobilis* CP4 (pZB206) strain was also constructed by introducing five arabinose metabolism- related genes from *E. coli* into *Z. mobilis* CP4 in 1996, which could ferment arabinose sugar and produced ethanol at 98% of theoretical yield [77]. For co-fermenting glucose, xylose, and arabinose to ethanol simultaneously, co-culture processes of *Z. mobilis* ATCC 39676 (pZB4L) and ATCC 39676 (pZB206) have been performed, which showed 72.5% of theoretical ethanol yield [78]. However, both xylose-fermenting strain and xylose had a significant effect on the performance of the arabinose utilization strain. Based on these considerations, Zhang *et al.* constructed a single *Z. mobilis* 206C (pZB301) in 1998, which could ferment mixture sugars to ethanol via 82 to 84% theoretical yield [79]. However, all recombinant strains were constructed by antibiotic-resistant plasmid; addition of antibiotics to maintain stablity for large-scale fermentations is highly undesirable. For enhancing its genetic stability, all seven genes necessary for pentose utilization were integrated into the*Zymomonas* genome and a stable *Z. mobilis* AX101 strain obtained in 2002, which could ferment a hextose and pentose mixture via a preferential order [80].

Although a strain capable of co-fermentation of all three sugars was achieved, all recombinant strains were sensitive to acetic acid stress. For example, nuclear magnetic resonance (NMR) studies found that acetic acid could inhibit efficiency of xylose utilization in *Z. mobilis* ZM4 (pZB5)[81]. Different strategies were developed to improve the tolerance of acetic acid and xylose utlization. For example, Lawford and Rousseau *et al.* developed a process via addition of extra glucose in acetic acid-containing media for improving fermentation performance of recombinant *Zymomonas*[82]. Recombinant plasmid pZB5 was also transferred into an acetic acid-tolerant strain (ZM4/Ac^R^) [47], and a mutant recombinant *Z. mobilis* ZM4/Ac^R^ (pZB5) strain with increased acetate resistance was obtained [48]. Overexpression of xylulokinase in a xylose-metabolising recombinant strain was also performed, and resulted in another recombinant ZM4/Ac^R^ (pZB5, pJX1) [83]. The ALE strategy was also used for improving the tolerance of acetic acid [69] and efficiency of xylose utilization [70] in *Z. mobilis* as mentioned previously. An isolated mutant CP4 (pZB5) M1-2 strain could metabolize xylose more rapidly than glucose. Sequence data analysis revealed mutations in both the glucose facilitator (*glf*) and glucokinase (*glk*) genes [84]. Mohagheghi *et al*. developed a new integrant of ZM4(pZB5), and named it*Z. mobilis* 8b, and this can tolerate acetic acid up to 16 g l^-1^ and achieve 82 to 87% ethanol yields [49]. Another mutant of *Z. mobilis* strain 8b obtained through adaptation using 2-deoxyglucose has shown a higher rate of xylose utilization [85]. Specific gene inactivation was also performed for strain improvement, for example, a superior strain, ZM6014 ^△^XR/pZMETX* obtained by inactivation of xylose reductase (*XR*, ZMO0976) [30,86]. Another example is *him*A (ZMO0976) inactive by transposon mutagenesis (as also shown in Table 1) [24,25]. In 2013, a cost-effective recombinant *Z. mobilis* HYMX was constructed by integrating seven genes (*Pfu-sHSP, yfdZ, metB, xylA, xylB, tktA* and *talB*) into the genome of *Z. mobilis* CP4 via Tn5 transposon, which showed tolerance tomultiple stresses, high yield and stable genetic characteristics [56].

Furthermore, fermentation characteristics of different recombinant strains were also analyzed in the past decade [30,49,56,78-81,83,84,86-89]. Importantly, fermentation performance of three best recombinant strains form different platforms used for cellulosic ethanol production, *E. coli* KO11, *S. cerevisiae* 424A (LNH-ST) and *Z. mobilis* AX101, which were compared with cellulosic material for the first time. Especially, *Z. mobilis* AX101 showed the highest rate of glucose consumption and lowest yield of byproducts [88]. These results also indicate that the metabolic pathway of *E. coli* KO11 and *Z. mobilis* AX101 are more effective in fermenting ethanol from the related yeast pathway of the consumed sugars [88]. However, utilization of xylose in lignocellulosic hydrolysate and growth robustness of recombinant *Z. mobilis*are also required to improve in the future. Moreover, different lignocellulosic feedstocks, such as agro-industrial wastes [90], sugarcane bagasse [91], oat hull [92], corn stover [49,93], bamboo residues [94], and various hydrolysates produced by Arkenol Technology [50], have also been used for ethanol production by *Z. mobilis*. In general, these studies will provide a deep basis for the ethanol industry in the future.

Although different engineered *Z. mobilis* strains have also been successfully constructed by introducing desirable genes as previously mentioned, convertion of cellulosic biomass into ethanol directly is also a considerable task for ethanol production. Recently, there has been development of consolidated bioprocessing (CBP)- a combination of cellulase production, cellulose hydrolysis and fermentation into a single step, which is regarded as an alternative approach with outstanding potential [95,96]. In 2010, two cellulolytic enzymes, E1 and GH12 from *Acidothermus cellulolyticus* were successfully expressed in *Z. mobilis* via a native secretion signal peptide [97]. Five cellulolytic enzymes from bacteria isolated from the gut of phytophagous insects were also transferred into *Z. mobilis*, and all the resulting recombinants fermented pretreated cellulosic feedstocks directly into ethanol [98]. In another study, six genes encoding cellulolytic enzymes (*CenA*, *CenB, CenD, CbhA, CbhB,* and *Cex*) from *Cellulomonas fimi* and other cellulolytic enzymes (*cenA*, *bgl*) from *Ruminococcus albus* were also introduced and co-expressed successfully in *Zymobacter palmae*, which enabled *Z. palmae* to efficiently ferment a water-soluble cellulosic polysaccharide to ethanol [99]. Although the recombinant *Z. mobilis* strains need to be improved further by simultaneous expression of additional cellulase genes, all these results also indicate that *Z. mobilis* could be serving as an important CBP platform organism.

### Other value-added bio-products production by *Z. mobilis*

#### *Sorbitol and bionic acid production*

In 2013, the US Department of Energy (DOE) published 12 topvalue-added building-block chemicals from biomass [100]. Representative chemicals, including four carbon 1,4-diacids (succinic, fumaric, and malic), 2,5-furan dicarboxylic acid (FDCA), 3-Hydroxypropionic acid (3-HPA), aspartic acid, glutamic acid, glucaric acid, itaconic acid, levulinic acid, 3-Hydroxybutyrolactone, glycerol, sorbitol, xylitol/arabinitol. Sorbitol was identified as one of the top 12 building block chemicals by the US DOE [100], and could be produced by *Z. mobilis*.

Actually, Barrow *et al.* found a phenomenon that ethanol yield was decreased when *Z. mobilis* grown on sucrose or mixtures of glucose plus fructose medium. Further study by NMR spectroscopy indicated that the reason for reduced ethanol yield was due to sorbitol formation from fructose [101]. Leigh *et al.* identified a proposed metabolic pathway for the production of sorbitol in *Z. mobilis*[102]. Zachariou and Scopes *et al.* demonstrated glucose-fructose oxidoreductase (GFOR) and glucono-σ-gluconase (GL) are responsible for sorbitol production, and gluconate intermediate could be converted to ethanol via the Entner-Doudoroff (ED) pathway [103]. These extensive studies demonstrated that *Z. mobilis* could produce sorbitol in a one-step reaction via GFOR, which is so far only known from this bacterium.

Based on these studies, many researchers developed different processes for producing sorbitol or gluconic acid production by *Z. mobilis* via whole cells, permeabilized cells or immobilized cells (as shown in Table 3). For example, Chun and Rogers *et al*. developed a simultaneous process for sorbitol and gluconic acid, 290 g/L of sorbitol and 283 g/L of gluconic acid were yielded from 60% total sugar solution (300 g L^-1^ glucose and 300 g L^-1^ fructose) after a 15-h reaction with *Z. mobilis*-permeabilized cells [104]. Rehr *et al.* found no gluconic acid formation when using glucose-grown cells for the conversion of equimolar fructose and glucose mixtures. However, nearly 295 g/L of sorbitol and gluconic acid were produced using cetyltrimethylammonium bromide (CTAB)-treated cells [105]. These results surported that gluconate intermediate converted to ethanol via the ED pathway [103,106]. Silveira *et al.* found that the yield of sorbitol and gluconic acid increased with substrate concentration [107]. Cazetta *et al.* investigated sorbitol production from sugar cane molasses by *Z. mobilis*, which showed the best conditions for sorbitol production containing 300 g/L total reducing sugars (TRS) in the culture medium [108]. Actually, to improve the sorbitol yield, various cell permeabilization methods, that is toluene [104], dried *Z. mobilis* cells, CTAB [105], metal ions [109], which inhibited key enzymes of the ED pathway and led to decreased ethanol concentration.

**Table 3:** **High yield of sorbitol and gluconic acid production by *Z. mobilis***

**Substrate**	**Biocatalyst**	**Products (g/L)**		**References**
**equimolar glucose plus fructose (g/L)**		**Sorbitol**	**Gluconic acid**	
600	Permeabilized cell	290	283	[104]
	Whole cell	240	ND	[105]
	Permeabilized cell	295	295	
100	Whole cell	12	1.5	[107]
300		105	50	
650		300	320	

Although *Lactobacillus casei*[110] and *Lactobacillus plantarum*[111] were also engineered for sorbitol production, sorbitol with a yield up to 0.65to 0.67 mol/mol glucose [111,112], the conversion rate of sugar and yield of sorbitol are lower when compared to *Z. mobilis.* So, *Z. mobilis* showed some advantages of sorbitol production, including a one-step reaction via GFOR, higher conversion rate of sugar and yield, and higher value of byproduct. It may be used for sorbitol production in an industrial scale in the future.

However, activity of GFOR in wild-type *Z. mobilis* is very low and regulated by glucose concentration [103]. For further improvement of sorbitol production, overexpression of GFOR is an attractive strategy to improve its efficiency. As reported by Liu *et al.*, an engineered strain harboring plasimd pHW20a-*gfor*, showed higher sorbitol yield than the wild strain [109]. On the other hand, although *Z. mobilis* could convert a mixture of glucose and fructose into sorbitol with high efficiency, the cost of the substrate may be very high. No natural feedstocks could meet the demand of high sugar-concentration. So, further research need to be carried out for searching for cheaper feedstocks or into the development of a novel process for conversion of lower sugar-concentration. Fortunately, the metabolic pathway of sorbitol and gluconic acid are clear [103,113], and gene regulation of *gfor* has also been studied by many research groups [32,114]. Loos *et al.* described a sorbitol-related protection mechanism of osmotic stress in concentrated sugar media [114]. Further research also indicates that sorbitol is required for cell growth and ethanol production under heat, ethanol, and osmotic stresses in *Z. mobilis*[32]. These clues will provide a chance for improving sorbitol and gluconic acid yield through metabolic engineering.

**Figure 3:**
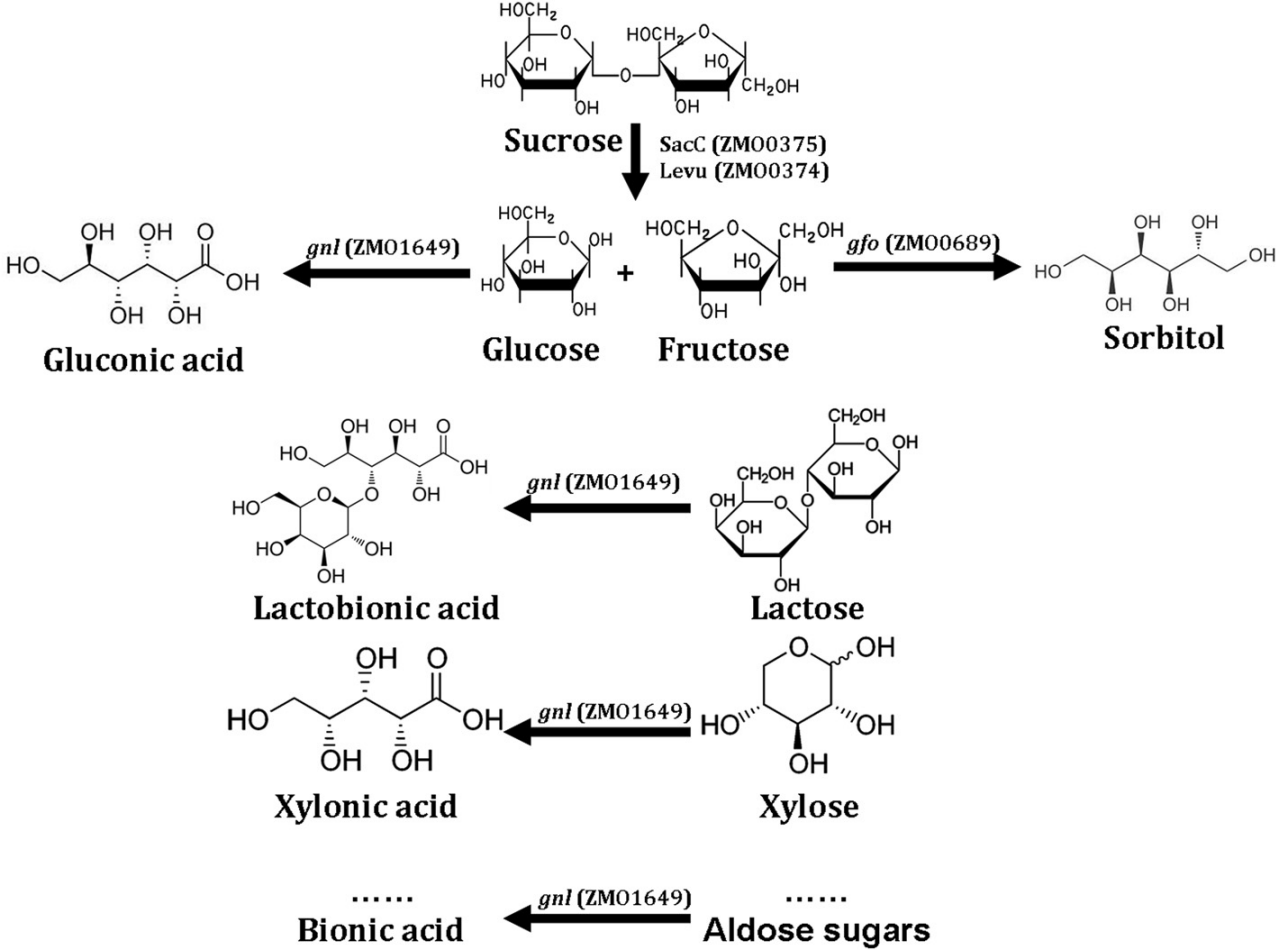
**Reaction scheme for the production of bionic acid and sorbitol via glucose-fructose oxidoreductase (GFOR) and glucono-σ-lactonase(GL) of *Z. mobilis.***

Furthermore, for determination of the substrate spectrum of GFOR, Satory *et al.* first reported that GFOR enzyme from *Z. mobilis* can oxidize different aldose sugars into corresponding aldonic acid when D-Fructose is used as the corresponding acceptor substrate. The conversion efficiency ranges from 9 to 84%, which shows a broad spectrum of substrates for the enzyme [115]. The study indicated that GFOR could be potentially used for other bionic acid production, that is, lactobionic acid (LBA), a lactose derivative that has many value-added applications in cosmetics, pharmaceutical or biomedicines, food, and chemical industries, as reviewed by Alonso *et al.*[113]. Lactose oxidation by GFOR was also performed by Satory *et al.*, which showed a high productivity of 110 g/L^-1^/d^-1^ in a continuously stirred tank reactor (CSTR) after operating for 70 h [115]. Bioconversion of a mixture of fructose and lactose into sorbitol and LBA with immobilized cells of *Z. mobilis* in calcium-alginate has also been reported [116,117]. Other bionic acids, such as maltobionic, xylonic acid, galactonic acid, arabinonic acid, mannonic acid and cellobionic acid, should also be performed in the future, which shows another important application for *Z. mobilis* (as shown in Figure 3).

#### *Levan production by Z. mobilis*

Levan is a fructose polymer with potential importance in food technology or medical applications [118]. Actually, Dawes and Ribbons *et al.* first found that reduction of ethanol yield has been attributed to levan formation when *Z. mobilis*is grown on sucrose medium [119]. Further research also verified that ethanol-yield reduction might be due to sorbitol and levan formation [101,102,120]. For example, Beker *et al.* developed a simultaneous sucrose bioconversion into ethanol and levan by *Z. mobilis*, and the levan yield of 0.22 g/g and the productivity of 3.2 g/L/h obtained [121]. Yoshida *et al.* and other researchers also found *Z. mobilis* could produce a high yield of levan when cultivated in sucrose medium [122-124]. Calazans *et al.* also found that levans produced by *Z. mobilis* strains have anti-tumor activities, and its molecular weight was also determined [125,126]. Previous studies verified that intracellular sucrase (*SacA*), extracellular levansucrase (*SacB*) and extracellular sucrase (*SacC*) contribute to sucrose hydrolysis in *Z. mobilis*[127]. Based on its genetic and biochemical studies, Senthilkumar *et al.* constructed a *SacC* mutant via the insertional mutant method, and higher yield of levan was obtained [20]. To avoid unnecessary supplementation with vitamins and mineral salts, low-cost effective substrate needs be used for levan production in *Z. mobilis*[128]. Levan production in batch and continuous fermentation systems by *Z. mobilis* B-14023 was also investigated recently [129]. These extensive studies indicate that *Z. mobils* may be used for industrial levan production for some purposes.

#### *Succinic acid production by Z. mobilis*

**Figure 4:**
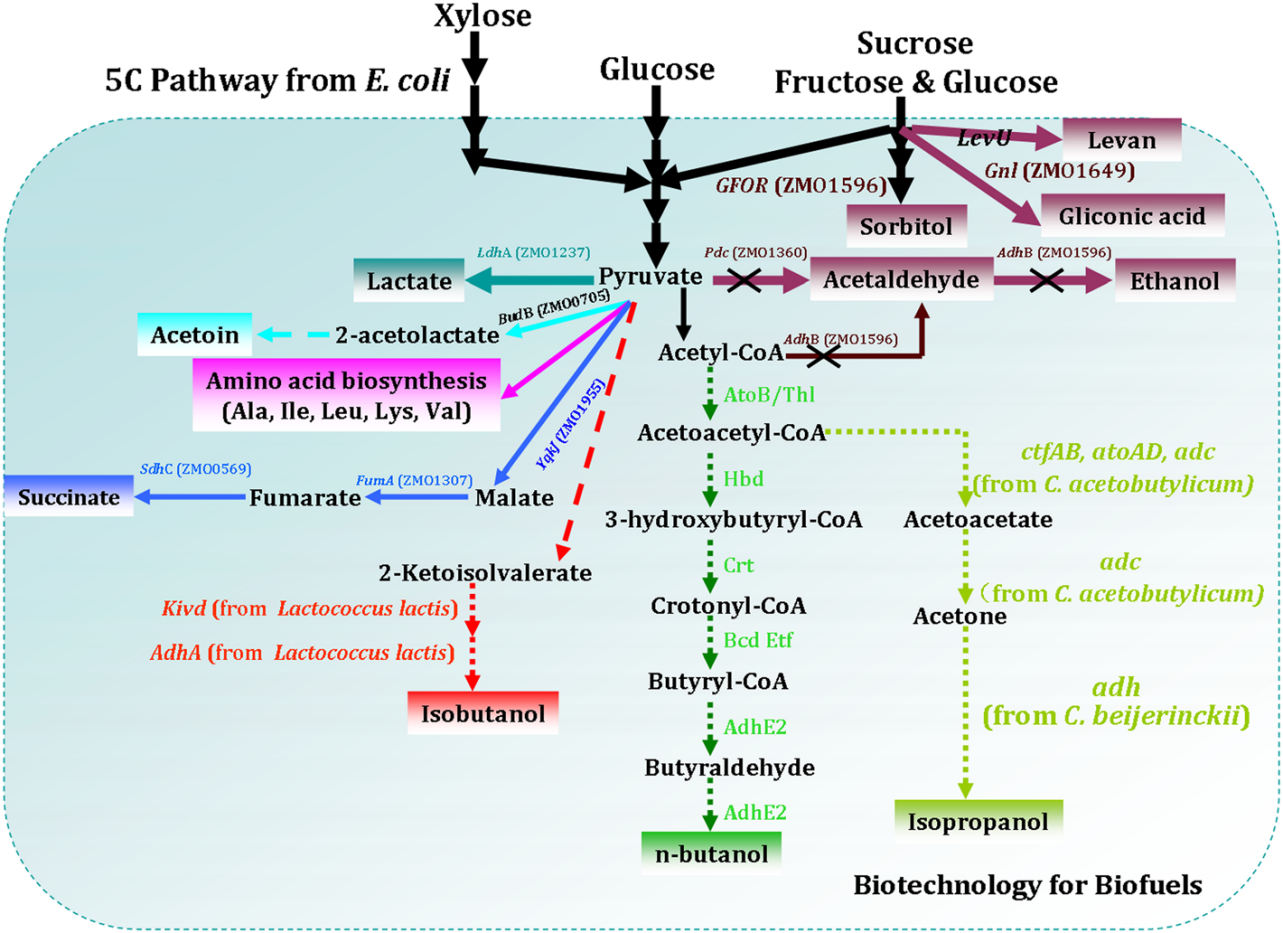
**Metabolic pathways for the production of the high-value products by using *Z. mobilis *as platform.** The solid lines indicate *Z. mobilis* native pathways and the dotted lines refer to the recombinant pathway obtained by metabolic engineering strategies. gfor, glucose-fructose oxidoreductase; *ldh*A, lactate dehydrogenase; pdc, pyruvate decarboxylase; *gnl*, glucono-σ-gluconase; *adc*, acetoacetate dehydrogenase; *adh*, secondary alcohol dehydrogenase; *adh*B, alcohol dehydrogenase; *adh*E, acetaldehyde/alcohol dehydrogenase; *adh*E2, secondary alcohol dehydrogenase; *ato*AD, acetyl-CoA:acetoacetyl-CoA transferase; *ato*B, acetyl-CoA acyltransferase; *bcd*, butyryl-CoA dehydrogenase; *crt*, crotonase; *ctf*AB, acetoacetyl-CoA transferase; *etf*BA, electrotransfer flavor protein; *hbd*, β-hydroxy butyryl-CoA dehydrogenase; *thl*, acetyl-CoA acyltransferase; *kivd*, ketoisovalerate decarboxylase.

Succinic acid was identified as one of the top 12 building-block chemicals by the US DOE[100]. Transparency Market Research also published a new report,*Succinic Acid Market - Global Industry Analysis, Size, Share, Growth, Trends and Forecast, 2012-2018,* in October 2013, which predicted that its market will be expected to reach USD 836.2 million by 2018. Based on these considerations, biological production of succinic acid from abundant and available biomass has become a topic of worldwide interest. Currently, different natural succinate-producing or genetically modified strains, such as *Actinobacillus succinogenes*, *Anaerobiospirillum succiniciproducens, Mannheimia succiniciproducens*, *Bacteroides fragilis, and Corynebacterium sp*. have been used for bio-based succinic acid production from different feedstocks [130,131]. Other strains, including *E. coli*[132,133], and *S. cerevisiae*[134,135] have also been engineered for succinic acid production. Although these strains showed some advantanges for succinic acid production, the process of fermentation is anaerobic and kinds of byproducts are formed. Recently, Lee *et al*. constructed a genome-scale metabolic model of *Z. mobilis* (ZmoMBEL601), which suggested a possible strategy for succinic acid production by disrupting pyruvate decarboxylase (*pdc*, ZMO1360) or alcohol dehydrogenase (*adh*B, ZMO1596) and D-lactate dehydrogenase (*ldh*A, ZMO1237) simultaneously [136]. Although this conclusion is based on the metabolic model, the higher yield of succinic acid will likely be achieved in the future. Actually, Seo *et al.* have constructed an engineered *Z. mobilis* for succinic acid production by redirecting metabolic pathways upon gene knockout of *pdc* and *ldh*A. The double gene-knockout strain ZM4 (^△^*pdc*^△^*ldh*A) has produced 1.46 mol succinate from 1 mol glucose, which showed 95% theoretical yield, and agrees well with the metabolic model ZmoMBEL601 [23]. Based on these studies, a suggested pathway for succinic acid may be proposed, as shown in Figure 4.

Other studies in silico or stoichiometric analysis of the central metabolism of *Z. mobilis*are valuable, for instance, Widiastuti *et al.* have also confirmed the functional role of *pdc* and *adh* genes during ethanol production in *Z. mobilis* via a genome-scale metabolic network (*i*zm363) [137]. A medium-scale model based on stoichiometric analysis of central metabolism was also performed by Pentjuss *et al.*[138]. These studies will also help us to gain a deep understanding of its special physiological characteristics or re-direct its metabolic pathway for production of target products in the future.

#### *Isobutanol production*

Isobutanol has also been paid more and more attention in recent years for its advantanges over bio-ethanol as a liquid fuel [2,139]. Engineered strains for isobutanol production in *E. coli*[139-141], *S. cerevisiae*[142-144], *Corynebacterium glutamicum*[143,145-147], *Bacillus subtilis*[148], and fungal-bacterial consortia [149], have been engineered or reviewed in previous studies. Recently, an engineered *Z. mobilis* strain was also constructed for isobutanol production via metabolic pathway engineering: 2-ketoisovalerate decarboxylase (*kiv*d) gene and alcohol dehydrogenase (*adh*A) gene from *Lactococcus lactis* were introduced into *Z. mobilis* ZM4, which led to isobutanol accumulation. Although the yield of isobutanol is very low, an engineered *Z. mobilis* is first used for producing the isobutanol. Higher yield may be obtained by disruption of key genes of the ED pathway (as shown in Figure 2) or addition of the extra biosynthesis pathway of alanine (for example, *alaD*, L-alanine dehydrogenase). Actually, *alaD* gene *Bacillus sphaericus* was also cloned and introduced into *Z. mobilis*, and 7.5 g/L alanine was excreted in the recombinant strain [150].

#### *Other products*

Isoprenoids represent another wide group of chemically active compounds, which could be produced by engineered microorganisms, and show a broad range of applications [151,152]. Actually, *Z. mobilis* has the highest total hopanoid content (30 mg/g DCW, dry cell weight) among all bacteria, which leads to more tolerance by increasing the hopanoid content [153,154]. Further research has also verified its biosynthesis pathway viathe methylerythritol phosphate (MEP) pathway [155]. Moreover, biosynthesis pathway of hopanoid lipids and its regulation have also been characterized on the genetic level, which formed a biosynthetic operon [43,44,156-158]. These results indicated that *Z. mobilis* has higher activity of the isoprenoid biosythensis pathway, which may be potentially used for isoprenoid compounds production and reflects a novel application for *Z. mobilis*. Actually, a group of plasmid-encoded carotene biosynthetic genes (*crtB*, *crtE*, *crtI*, *crtY*) have been introduced into *Z. mobilis* via conjugation, resulting in production of β-carotene [159]. Several genes from the thermallydimorphic fungus *Penicillium marneffei* with predicted terpene synthase function were also selected for functional analysis and evaluation of their potential for the bioproduction of isoprenoid compounds in *Z. mobilis* (as shown in the PhD thesis of So Lok-yan, University of Hong Kong). Although these studies represent preliminary work, with deeper understanding of its biosynthesis pathway, *Z. mobilils*shows great potential for isoprenoid compounds production in the future.

## Conclusions

**Figure 5:**
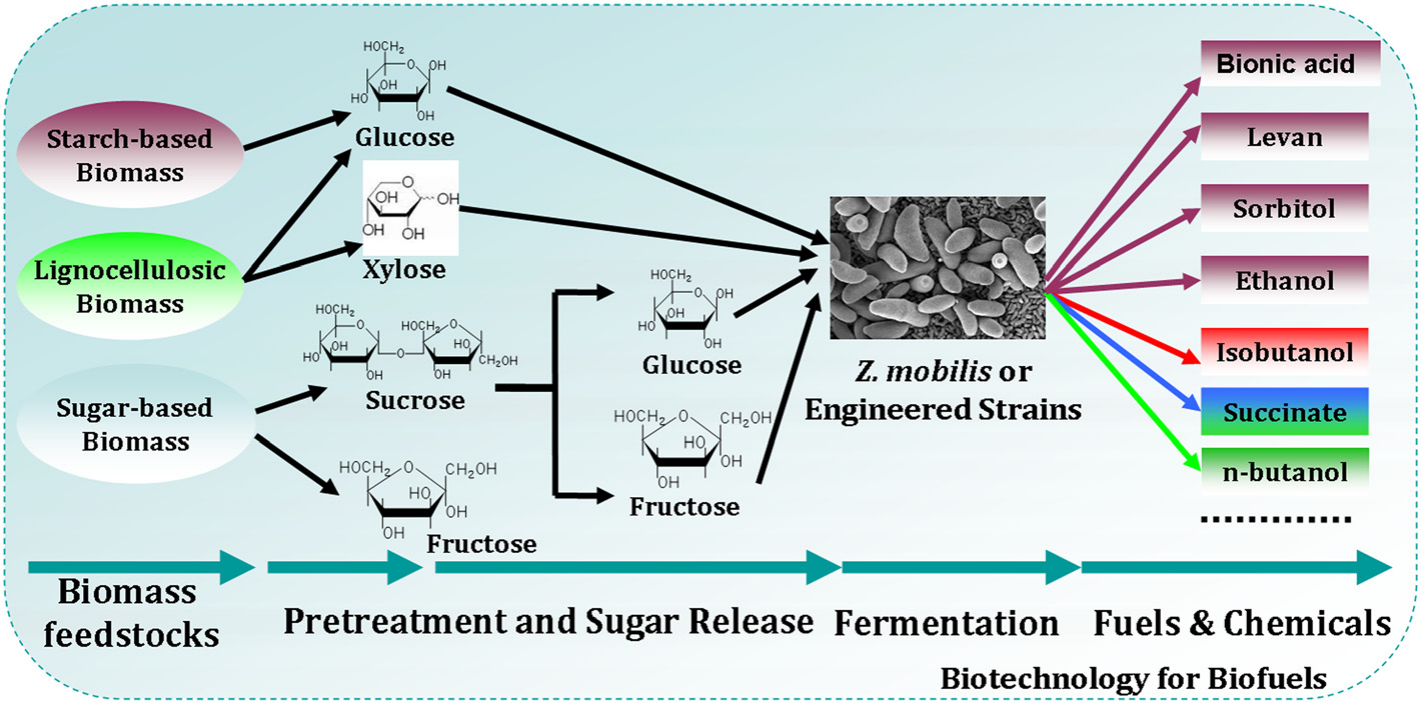
**General process of fuel or chemical production by *Z. mobilis*.**

Based on the previous and our reviews, *Z. mobilis* is firstly being developed as an effective ethanologenic by engineering strain improvement, including utilization of xylose and arabinose in addition to glucose. Undoubtedly, *Z. mobilis* has showed desirable characteristics for its special metabolic pathway. The scientific and technological progress of *Z. mobilis* have also made a significant contribution to the bioethanol industry. Compared with *E. coli*, *Z. mobilis* has high restriction-modification enzyme activity, and cannot be contaminated by bacteriphages [13]. It is fairly osmo-tolerant and can hence tolerate very high sugar concentrations, which is an advatange in fermentation in a high-sugar medium. Its smaller genome and simple metabolic pathway, also lead to less byproducts formation. On the other hand, its desirable characteristics will also make it a novel platform for future biorefineries, which will make a significant contribution to green or sustainable chemistry (as shown in Figure 5).

Although extensive studies, such as general genetic tools, strategies of metabolic engineering, value-added bio-product production, genomic and transcriptomic, et cetera, have been developed in *Z. mobilis* since 1980s, non-commercialization of the *Zymomonas* process for ethanol production from sugar, starch-based or lignocellulosic biomass has developed successfully. Moreover, an increased range of higher-value product generation has also been restricted by its fundamental research. Especially, it is more difficult to engineere*Z. mobilis* than *E. coli* or yeast. Despite the extensive studies on general genetic tools and *omics* data available for *Z. mobilis*, it is necessary to further develop advanced technologies that can be used in metabolic engineering.

Therefore, to realize the industrial potential of *Z. mobilis* for future biorefineries, considerable efforts should be focused on the following points in the future: developing universal tools for deletion of several genes in one round, controlling metabolic flux and optimizing regulatory networks to improve the yield of desired products, and developing a highly express system, et cetera; these novel technologies are necessary for further strain improvement or redirection of the metabolic pathway for fuel and chemical production. Moreover, different systems of metabolic engineering approaches are becoming powerful tools in developing engineered *E. coli* or *S. cerevisiae*[3,5], which should also be highlighted in engineered *Z. mobilis* strains. In particular, other biotechnological approaches, such as genome sequencing, functional genomics, genome engineering and *omics* will also provide a basis for pathway or genome reconstruction to improve its fitness and robustness for environmental stress [160,161]. Representitive biotechnologies, such as CRISPR/Cas systems [162], site-specific recombinases [163,164], genome shuffling [165], global transcription machinery engineering (gTME) [166], and Zinc-finger nucleases [167], which will also be used for enhancing cellular traits of *Z. mobilils.* Presumably, their potential will be further implemented with a promising future in developing or optimizing the metabolic pathway for the production of fuels as well as commodity and specialty chemicals.

## Abbreviations

AcR: acetate-tolerant mutant; ALE: adaptive laboratory evolution; bp: base pairs; CBP: consolidated bioprocessing; CSTR: continuously stirred tank reactor; CTAB: cetyltrimethylammonium bromide; US DOE: US Department of Energy; ED pathway: Entner-Doudoroff pathway; GFOR: glucose-fructose oxidoreductase; GL: glucono-σ-lactonase; gTME: global transcription machinery engineering; LBA: lactobionic acid; NGS: next-generation sequencing; NMR: nuclear magnetic resonance; subsp.: subspecies.

## Competing interests

The authors declare that they have no competing interests.

## Authors’ contributions

This review was conceived, researched and written by MXH. BW and ZYR participated in *omic* data collection and helped in manuscript editing. FRT summarized the section on strain improvement for *Z. mobilis*. JLW, ZXS, HQ, and QLZ summarized the section on succinic acid production by *Z. mobilis* and helped in management of references. LCD, XYT, and WGW participated in data collection and were involved in drafting the manuscript. KP and QCH participated in the discussion and helped in the draft manuscript editing. All authors read and approved the final manuscript.
